# Herring Oil and Omega Fatty Acids Inhibit *Staphylococcus aureus* Biofilm Formation and Virulence

**DOI:** 10.3389/fmicb.2018.01241

**Published:** 2018-06-15

**Authors:** Yong-Guy Kim, Jin-Hyung Lee, Chaitany J. Raorane, Seong T. Oh, Jae G. Park, Jintae Lee

**Affiliations:** ^1^School of Chemical Engineering, Yeungnam University, Gyeongsan, South Korea; ^2^College of Pharmacy, Yeungnam University, Gyeongsan, South Korea; ^3^Advanced Bio Convergence Center, Pohang Technopark Foundation, Pohang, South Korea

**Keywords:** biofilm, hemolysis, herring oil, omega fatty acids, *Staphylococcus aureus*, virulence

## Abstract

*Staphylococcus aureus* is notorious for its ability to become resistant to antibiotics and biofilms play a critical role in antibiotic tolerance. *S. aureus* is also capable of secreting several exotoxins associated with the pathogenesis of sepsis and pneumonia. Thus, the objectives of the study were to examine *S. aureus* biofilm formation *in vitro*, and the effects of herring oil and its main components, omega fatty acids [*cis*-4,7,10,13,16,19-docosahexaenoic acid (DHA) and *cis*-5,8,11,14,17-eicosapentaenoic acid (EPA)], on virulence factor production and transcriptional changes in *S. aureus*. Herring oil decreased biofilm formation by two *S. aureus* strains. GC-MS analysis revealed the presence of several polyunsaturated fatty acids in herring oil, and of these, two omega-3 fatty acids, DHA and EPA, significantly inhibited *S. aureus* biofilm formation. In addition, herring oil, DHA, and EPA at 20 μg/ml significantly decreased the hemolytic effect of *S. aureus* on human red blood cells, and when pre-treated to *S. aureus*, the bacterium was more easily killed by human whole blood. Transcriptional analysis showed that herring oil, DHA, and EPA repressed the expression of the α-hemolysin *hla* gene. Furthermore, in a *Caenorhabditis elegans* nematode model, all three prolonged nematode survival in the presence of *S. aureus*. These findings suggest that herring oil, DHA, and EPA are potentially useful for controlling persistent *S. aureus* infection.

## Introduction

The sticky conglomerations of bacteria that adhere to diverse medical devices or damaged body tissues can become causes of persistent infections. These bacteria encase themselves in a slime layer or biofilm, which poses serious problems to human health because of their abilities to tolerate conventional antibiotic chemotherapies, host immune systems, and external stresses (Potera, 1999; Hoffman et al., 2005).

*Staphylococcus aureus* is an important etiologic agent and a major cause of a diverse array of acute life-threatening bloodstream infections in man, and is often held responsible for worldwide outbreaks of nosocomial infections (Lowy, 1998). *S. aureus* biofilms play a critical role in antibiotic tolerance (Stewart and Costerton, 2001), and methicillin-resistant *S. aureus* (MRSA) and vancomycin-resistant *S. aureus* have become major nosocomial threats (Speller et al., 1997). *S. aureus* secretes many exotoxins, including coagulase, enterotoxins, α-hemolysin, protein A, and TSST-1, which damage biological membranes and eventually cause cell death (Ohlsen et al., 1997; Otto, 2014). Also, *S. aureus* biofilms are found in medical devices and food surfaces, and are responsible for food-poisoning and toxic shock syndrome (Chambers and Deleo, 2009). In particular, α-hemolysin (Hla) is a major cytotoxic agent that has been associated with the pathogeneses of pneumonia, sepsis, and severe skin infections (Menzies and Kernodle, 1996; Wilke and Bubeck Wardenburg, 2010) and with biofilm formation (Caiazza and O’Toole, 2003). *S. aureus* biofilms also play a dominant role in the determination of disease severity and postoperative course (Singhal et al., 2011). Therefore, it is important to find means to inhibit biofilm formation and the virulent characteristics of *S. aureus.* For this purpose, novel and non-toxic compounds that prevent the development of drug tolerance are urgently required.

Semi-dried raw Pacific herring (*Clupea pallasi*), also known as Gwamegi (a Korean traditional food), is an important fishery product with a unique flavor, taste, and texture (Lee et al., 2002; Kang et al., 2011). Herring oil contains an abundance of two rare omega-3 polyunsaturated fatty acids (PUFAs), namely, docosahexaenoic acid (DHA; C22:6, ω-3) and eicosapentaenoic acid (EPA; C20:5, ω-3), which are commonly present at low concentrations in non-marine animals and are beneficial to the human body (Hirai et al., 1980; Schmidt and Dyerberg, 1994). Furthermore, it has been reported that two PUFAs act as bacteriocides on Gram-positive and Gram-negative bacteria, such as, *Helicobacter pylori* (Correia et al., 2012), *Burkholderia cenocepacia* (Mil-Homens et al., 2012), *S. aureus* (Desbois et al., 2009), *Fusobacterium nucleatum, Porphyromonas gingivalis*, and *Streptococcus mutans* (Huang and Ebersole, 2010; Sun et al., 2016, 2017). These two PUFAs have also been reported to possess significant anti-inflammatory, anti-tumorigenic (Bougnoux, 1999; Van Dyke, 2008), and antioxidant activities (Giordano and Visioli, 2014). Huang and Ebersole (2010), Sun et al. (2016, 2017) and Thibane et al. (2010) suggested that DHA and EPA could be considered as potential supplementary therapeutic agents due to their anti-biofilm activities on *Candida* species and periodontopathic bacteria. However, the abilities of DHA and EPA to inhibit biofilm formation and virulence production by *S. aureus* have not been assessed.

Therefore, the phenotypic effects of herring oil were studied and its active constituents were identified by gas chromatography-mass spectrometry (GC-MS). Two of its major constituents (DHA and EPA) were further investigated by confocal laser scanning microscopy (CSLM) and using a human blood assay to determine their effects on biofilm formation and toxin production by *S. aureus*. A *Caenorhabditis elegans* model was used to investigate the anti-virulent properties of herring oil, DHA, and EPA. qRT-PCR (quantitative real-time reverse transcription polymerase chain reaction) was used to investigate their effects on the transcriptional profiles of genes related to biofilm formation and virulence production *in vitro*.

## Materials and Methods

### Ethics Statement

Human blood assays were authorized by the Ethical Committee of Yeungnam University (Gyeongsan, South Korea). The study was conducted according to the guidelines issued by the Ethical Committee of Yeungnam University. All blood donors provided written consent before blood collection.

### Bacterial Strains, Materials, and Growth Assay

A methicillin-sensitive *S. aureus* strain (MSSA; ATCC 6538) and a methicillin-resistant *S. aureus* strain (MRSA; ATCC 33591) were used in the present study. All experiments were conducted at 37°C in Luria-Bertani (LB) broth for the MSSA strain and in LB broth containing 0.2% glucose for the MRSA strain. Herring (*Clupea pallasii*) oil was prepared from semi-dried fish bodies without organs (so called Gwamegi, Korean traditional food). Herring was dried at open air condition for 20 days in winter at Pohang, Korea. The semi-dried herring (764 g) was further dried in a freezing drier for 3 days to gain 586 g of dried herring. Then it was chopped, and the oil was extracted by soxhlet apparatus that was refluxed with n-hexane for a day at 70°C. After refluxing, the residual solution was collected and washed with 1% AcOH solution and brine to remove excess protein. Washed oil was dried over anhydrous MgSO_4_ to gain clear yellow viscous oil (267 g, 45.6% yield). The omega fatty acids (*cis*-4,7,10,13,16,19-DHA, *cis*-5,8,11,14,17-EPA, *cis*-11-eicosenoic acid, and erucic acid), crystal violet, ethanol, ciprofloxacin, vancomycin and glucose were purchased from Sigma-Aldrich (St. Louis, MO, United States). For cell growth assays, optical densities were measured at 600 nm using a spectrophotometer (Optizen 2120UV, Mecasys, South Korea). To determine MIC (minimum inhibitory concentration), cells were inoculated into LB broth and cultured overnight at a dilution of 1:100 at 37°C, and then incubated for 24 h in the presence of a test substance. After sequential dilutions, cultures were spread on LB agar plates, incubated for 24 h at 37°C, and numbers of colonies were counted. Each experiment was performed using at least four independent cultures.

### Gas Chromatograph/Mass Spectroscopy (GC-MS)

The chemical composition of air-dried herring oil was determined by GC using an Agilent 6890N GC and SP-2560 (Supelco, Sigma-Aldrich, St. Louis, MO, United States) fused silica capillary column (100 m × 0.25 mm i.d., film thickness 0.25 μm). Capillary column details, temperature conditions, and the derivatization (methylation) of fatty acids were as previously described (Lee et al., 2017). In brief, helium was used as the carrier at 0.75 ml/min and the GC injector was held at 225°C. The GC oven temperature was programmed as follows; 100°C for 4 min, increased to 240°C at 3°C/min, and followed by 15 min at 250°C. The split ratio was controlled at 200:1. Triundecanoin (C_11:00_) was used as the internal standard and quantifications were performed by integrating areas and correcting for fatty acid methylation. Supelco 37 components FAME Mix (Supelco) were used as the reference standard.

### Crystal-Violet Biofilm Assay

The two bacterial strains (MSSA 6538, MRSA 33591) were subjected to a static biofilm formation assay on 96-well polystyrene plates (SPL Life Sciences, South Korea), as previously described (Kim et al., 2015). Briefly, cells were inoculated into LB broth (300 μl) at an initial turbidity of 0.05 at 600 nm and herring oil, *cis*-11-eicosenoic acid, DHA, EPA, or erucic acid were added at different concentrations and incubated for 24 h without shaking at 37°C. To quantify biofilm formation, cell cultures were washed three times with water and then biofilms were stained with 0.1% crystal violet for 20 min. Biofilms were then dissolved in 300 μl of 95% ethanol and absorbances were measured at 570 nm (OD_570_). For the biofilm dispersion assay, *S. aureus* was cultured in 96-well plates for 24 h without shaking at 37°C. Then, herring oil, DHA, or EPA was added to the cultures and incubated for another 10 h before the biofilm assay. Static biofilm formation results are the averages of four independent cultures of twelve replicate wells.

### Confocal Laser Scanning Microscopy and COMSTAT Analysis

Static biofilm formation by *S. aureus* (MSSA 6538) in 96-well plates (without shaking) in the presence or absence of herring oil, DHA, or EPA were assessed by confocal laser scanning microscopy (Nikon Eclipse Ti, Tokyo, Japan). Cells were stained with carboxyfluorescein diacetate succinimidyl ester (Invitrogen, Molecular Probes Inc., Eugene, OR, United States), which stains viable cells in biofilms, as previously reported (Lee et al., 2016). Stained *S. aureus* ATCC 6538 biofilms were visualized by confocal laser scanning microscopy using an Ar laser (excitation wavelength 488 nm, and emission wavelength 500–550 nm) and a 20× objective. Color confocal images were constructed using NIS-Elements C version 3.2 (Nikon eclipse) under the same conditions. For each experiment, at least 10 random positions in two independent cultures were observed, and 20 planar images were analyzed per position. To quantify biofilm formation, COMSTAT biofilm software (Heydorn et al., 2000) was used to measure biovolumes (μm^3^ per μm^2^), mean thicknesses (μm), and substratum coverages (%). Thresholds were fixed for all image stacks, and at least 4 positions and 20 planar images were analyzed per position.

### Hemolysis Assay

Human red blood cell hemolysis efficacies were assayed using whole cultures of *S. aureus*, as described previously (Lee et al., 2013; Tharmalingam et al., 2018). Briefly, *S. aureus* cells (MSSA 6538) were diluted 1:100 in LB broth (9 × 10^9^ CFU/ml) with overnight culture and incubated with or without herring oil, DHA, or EPA for 20 h with shaking at 250 rpm. Separately, human whole blood samples were centrifuged at 900g for 2 min and then washed three times with PBS and diluted in PBS (330 μl of red blood cells per 10 ml of PBS buffer). Bacterial culture (200 μl) was then added to 1 ml of diluted human red blood cells (3.3% in PBS). To determine hemolytic activities, mixtures of blood and *S. aureus* were incubated at 250 rpm for 2 h at 37°C. Supernatants were collected by centrifugation at 16,600 *g* for 10 min and optical densities were measured at 543 nm.

### Whole Blood Bacterial Cell Killing Assay

The whole blood cell killing assay used was as previously described (Liu et al., 2008). Briefly, *S. aureus* (MSSA 6538) cells were inoculated (1:100 dilution, OD_600_ ∼0.05) in LB broth with overnight culture and incubated at 37°C for 20 h with shaking at 250 rpm with herring oil, DHA, or EPA (1, 5, or 20 μg/ml) or DMSO (the negative control). Freshly drawn human whole blood (0.3 ml) was then mixed with the *S. aureus* cultures (0.1 ml), and mixtures were incubated at 37°C for 3 h with shaking at 250 rpm. *S. aureus* (MSSA 6538) survival was measured by counting CFUs on LB agar plates.

### *Caenorhabditis elegans* Survival Assay

To investigate the effects of herring oil and unsaturated fatty acids on the virulence of *S. aureus* MSSA 6538, we used a nematode survival assay as previously described (Kim et al., 2016) with slight modification. In brief, *S. aureus* cells were incubated with or without herring oil, DHA, or EPA (2, 5, or 20 μg/ml) at 37°C for 24 h and synchronized adult *C. elegans fer-15;fem-1* nematodes were added into single wells of 96-well plate containing cultivated *S. aureus* cells. Approximately, 30 nematodes were allowed to feed on the cultured *S. aureus* MSSA 6538 at 25°C for 1 day.

For the cytotoxicity assay, 110 ± 10 nematodes were added into single well of 96-well plates containing M9 buffer and solutions of the compounds were added to final concentrations of 20 or 100 μg/ml at 25°C for 1 day. Then, nematodes were scored as alive or dead using an iRiS^TM^ Digital Cell Imaging System (Logos Bio Systems, South Korea). At least three independent experiments were conducted using quadruplicate wells.

### RNA Isolation and qRT-PCR

*Staphylococcus aureus* MSSA 6538 cells were inoculated into 25 ml of LB broth at 37°C in 250 ml flasks at a starting OD_600_ of 0.05, and then incubated for 5 h with shaking at 250 rpm in the presence or absence of herring oil (100 μg/ml), DHA, or EPA (20 μg/ml). RNase inhibitor (RNAlater, Ambion, TX, United States) was then added and cells were immediately chilled for 30 s in dry ice bath having 95% ethanol to prevent RNA degradation. Cells were then harvested by centrifugation at 16,600 *g* for 1 min and total RNA was isolated using a Qiagen RNeasy mini Kit (Valencia, CA, United States).

Quantitative real-time reverse transcription polymerase chain reaction was used to investigate the transcriptional levels of 10 biofilm-related genes, that are, *agrA*, *arlR*, *arlS*, *aur*, *hla*, *icaA*, *nuc1*, *rbf*, *RNAIII*, *saeR*, *sarA*, *sarZ*, *seb*, *sigB*, and *spa* in *S. aureus* MSSA 6538 cells. Gene specific primers were used and 16s rRNA was used as the housekeeping control (Supplementary Table S1) to normalize the expressions of genes of interest. The qRT-PCR technique employed was an adaptation of a previously described method (Lee et al., 2016). qRT-PCR was performed using a SYBR Green master mix (Applied Biosystems, Foster City, CA, United States) and an ABI StepOne Real-Time PCR System (Applied Biosystems). Expression levels were determined using two independent cultures and six qRT-PCR reactions per gene.

### Statistical Analysis

Values were expressed as means ± standard deviations, and data were analyzed by one-way ANOVA followed by Dunnett’s test using SPSS version 23 (SPSS Inc., Chicago, IL, United States) to determine the significances of differences. Statistical significance was accepted for *p*-values of <0.05.

## Results and Discussion

### Effect of Herring Oil on *S. aureus* Biofilm Formation

**FIGURE 1:**
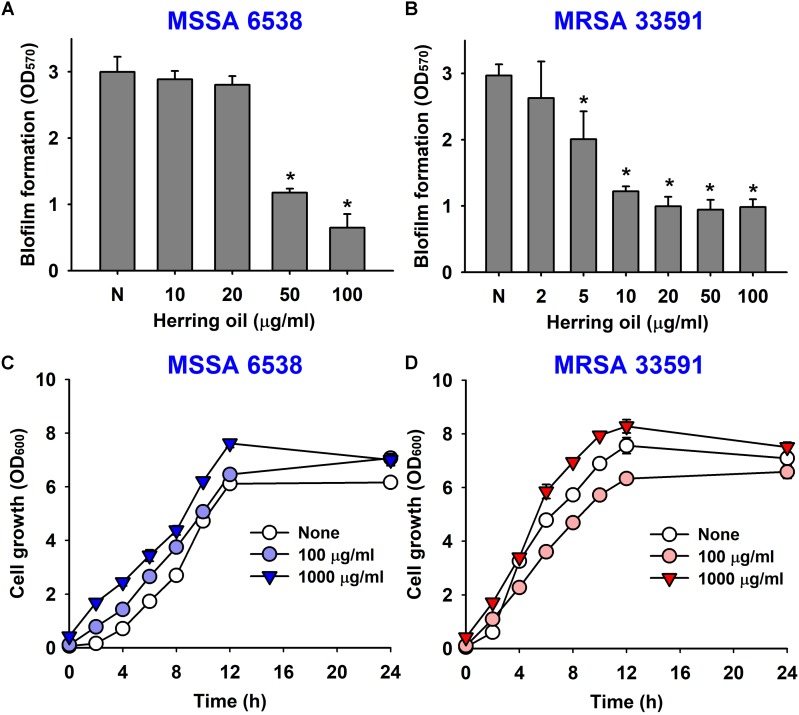
Effects of herring oil on biofilm formation by *Staphylococcus aureus* strains and on their antimicrobial activities. *S. aureus* (MSSA 6538 or MRSA 33591) biofilm formations (OD_570_) were quantified in the presence of herring oil for 24 h in 96-well polystyrene plates **(A,B)**. Planktonic cell growths (OD_600_) of *S. aureus* cells (MSSA 6538 and MRSA 33591) were measured in the presence of herring oil in 250 ml flasks agitated at 250 rpm **(C,D)**. ^∗^*p* < 0.05 vs. non-treated controls.

The anti-biofilm activity of herring oil was investigated against two *S. aureus* strains (MSSA 6538 and MRSA 33591) in 96-well polystyrene plates. The addition of herring oil at the beginning of bacterial culture dose-dependently inhibited *S. aureus* biofilm formation (**Figures 1A,B**). Specifically, herring oil at 100 μg/ml reduced biofilm formation by MSSA 6538 by >75%, whereas 20 μg/ml was required to inhibit biofilm formation by MRSA 33591 by >65%.

Herring oil did not decrease the growth of *S. aureus* strains (MSSA 6538 and MRSA 33591) at concentrations up to 1000 μg/ml (**Figures 1C,D**), and did not affect planktonic cell numbers of either *S. aureus* strain at concentrations up to 1000 μg/ml (data not shown). These findings show the anti-biofilm activity of herring oil was not due to any bactericidal effects.

### Identification of Main Components in Herring Oil

Gas chromatography-mass spectrometry identified 28 fatty acids in herring oil (**Table 1**). Components that constituted more than 5% of herring oil were; erucic acid (17.49%), *cis*-4,7,10,13,16,19-DHA (11.31%), *cis*-11-eicosenoic acid (9.75%), palmitic acid (8.18%), myristic acid (5.78%), and *cis*-5,8,11,14,17-EPA (5.78%) (**Table 1**). The compositions of herring oil identified by GC-MS analysis concur with previous studies (Lambertsen and Braekkan, 1965). Previously, unsaturated fatty acids, such as, *cis*-11-eicosenoic acid and oleic acid showed strong anti-biofilm effect while saturated fatty acids (myristic acid and palmitic acid) did not have anti-biofilm activity against *S. aureus* (Lee et al., 2017). Therefore, this study was focused on the anti-biofilm activities of three unsaturated predominant omega fatty acids (DHA, EPA, and erucic acid).

**Table 1:** Herring oil – GC-MS analysis.

Lipid number	Fatty acid name	Composition (%)
C12:0	Lauric acid	0.13
C13:0	Tridecanoic acid	0.03
**C14:0**	**Myristic acid**	**5.78**
C14:1	Tetradecenoic	0.05
C15:0	Pentadecanoic acid	0.45
**C16:0**	**Palmitic acid**	**8.18**
C16:1	Palmitoleic acid	2.15
C17:0	Heptadecanoic acid	0.36
C17:1	*cis*-10-Heptadecenoic aid	0.05
C18:0	Stearic acid	1.35
C18:1n-9,trans	Elaidic acid	0.09
C18:1n-9,cis	Oleic acid	2.72
C18:2n-6,cis	Linoleic acid	1.11
C18:3n-6	γ-Linolenic acid	0.20
**C20:1**	***cis*-11-Eicosenoic acid**	**9.75**
C18:3n-3	α-Linolenic acid	1.23
C21:0	Heneicosanoic acid	0.22
C20:2n-6	Eicosadienoic acid	3.80
C22:0	Behenic acid	0.05
C20:3n-6	Dihomo-γ-Linolenic acid	0.07
**C22:1n-9**	**Erucic acid**	**17.49**
C20:3n-3	*cis*-11,14,17-Eicosatrienoic acid	0.1
C23:0	Tricosanoic acid	0.24
C22:2	Docosadienoic acid	0.91
C24:0	Lignoceric acid	0.02
**C20:5n-3**	***cis*-5,8,11,14,17-Eicosapentaenoic acid (EPA)**	**5.78**
C24:1	Nervonic acid	0.93
**C22:6n-3**	***cis*-4,7,10,13,16,19-Docosahexaenoic acid (DHA)**	**11.31**

*Components present at levels >5% by weight are shown in blue/bold font.*

### Effect of DHA and EPA on Biofilm Formation by *S. aureus*

**FIGURE 2:**
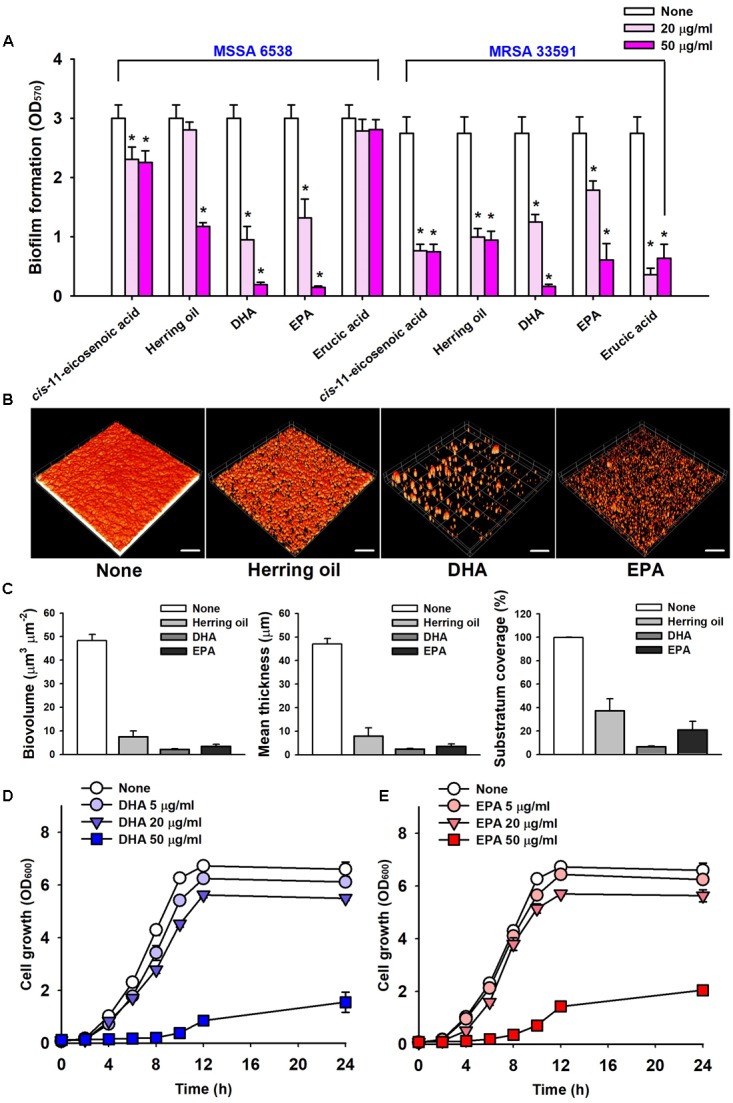
Inhibitions of biofilm formation by herring oil, DHA, and EPA. Biofilm formations by the two *S. aureus* strains were quantified in the presence of *cis*-11-eicosenoic acid, herring oil, DHA, EPA, and erucic acid at 20 or 50 μg/ml after incubation for 24 h in 96-well polystyrene plates without agitation **(A)**. Biofilm formation of *S. aureus* MSSA 6538 was observed after 24 h in the presence of herring oil (50 μg/ml), DHA (20 μg/ml), or EPA (20 μg/ml) in 96-well plates by confocal laser scanning microscopy **(B)**. The scale bars represent 100 μm. Biofilm formation was analyzed using COMSTAT **(C)**. Cell growth of *S. aureus* MSSA 6538 was measured in the presence of DHA or EPA **(D,E)**. ^∗^*p* < 0.05 vs. non-treated controls.

The anti-biofilm potencies of four major PUFAs (*cis*-11-eicosenoic acid, DHA, EPA, and erucic acid) were tested against the two *S. aureus* strains (MSSA 6538 and MRSA 33591) in 96-well plates. *cis*-11-Eicosenoic acid, DHA, and EPA at concentrations of 20 μg/ml significantly inhibited biofilm formation by both *S. aureus* strains (**Figure 2A**). DHA and EPA both inhibited biofilm formation by >90% at 50 μg/ml, which was greater than that previously reported for *cis*-11-eicosenoic acid (Lee et al., 2017). In this previous report, relationships between free fatty acids and bacterial biofilm formation were studied. In particular, oleic acid and *cis*-2-decenoic acid were found to suppress biofilm formation of *S. aureus* by blocking bacterial adhesion and dispersion from established biofilms, respectively (Stenz et al., 2008; Davies and Marques, 2009). In addition, it has been suggested that long chain unsaturated fatty acids like *cis*-11-eicosenoic acid (Lee et al., 2017) inhibit the biofilm forming ability of *S. aureus*. However, the present study is the first to report the anti-biofilm activities of DHA and EPA against *S. aureus*. The unsaturated fatty acid erucic acid, which was present in herring oil at high levels (**Table 1**), at 20 μg/ml inhibited biofilm formation by MRSA 33591 by >85%, but did not inhibit biofilm formation by MSSA 6538 (**Figure 2A**). However, higher concentrations of erucic acid at 500 and 1000 μg/ml significantly reduced biofilm formation of MSSA 6538 without affecting planktonic growth (data not shown). Hence, it appears that the effects of erucic acid are dose-dependent and strain dependent.

Confocal laser scanning microscopy was used to quantify biofilm formation by *S. aureus* in the presence of herring oil, DHA, or EPA. Fluorescent stacking 3D images indicated that herring oil (50 μg/ml), DHA (20 μg/ml), or EPA (20 μg/ml) markedly inhibited *S. aureus* biofilm formation (**Figure 2B**), and this, was confirmed by COMSTAT. More specifically, DHA, EPA, and herring oil reduced all three *S. aureus* MSSA 6538 parameters measured (biovolume, mean thickness, and substratum coverage) (**Figure 2C**), actually, DHA and EPA reduced biomass and mean thickness by >90%, respectively. These results show herring oil, DHA, and EPA all effectively reduced biofilm formation on the bottom of 96-well plate.

Mature biofilm detachment by antibiotics, enzymes, and fatty acids is interest due to their resistance to antimicrobial agents (Flemming and Wingender, 2010; Gwisai et al., 2017; Lee et al., 2018). However, herring oil (1000 μg/ml), DHA (100 μg/ml), and EPA (100 μg/ml) could not disperse matured *S. aureus* ATCC 6538 biofilms (data not shown).

### Anti-microbial Effects of DHA and EPA on *S. aureus*

The anti-microbial activities of DHA and EPA were evaluated by determining their minimum inhibitory concentrations (MICs) against *S. aureus* MSSA 6538 and MRSA 33591. Planktonic growth was completely inhibited by DHA and EPA at MICs (200 μg/ml), while MICs of ciprofloxacin and vancomycin are 0.5–1 μg/ml and 4 μg/ml, respectively, which concurs with previously reported values (Desbois and Lawlor, 2013; Gwisai et al., 2017). Notably, these MICs of DHA and EPA were 10 times higher than the concentration (20 μg/ml) required for anti-biofilm activities (**Figure 2A**).

Planktonic cell growths of *S. aureus* MSSA 6538 and MRSA 33591 in the presence of 5 μg/ml DHA or 20 μg/ml EPA were slightly inhibited (**Figures 2D,E**). However, the cell growths of both strains were more than 68% suppressed by DHA or EPA at 50 μg/ml, which indicates both DHA and EPA have anti-bacterial effects at relatively high concentrations (**Figures 2D,E**). These observations suggest DHA and EPA at low concentrations have anti-biofilm effects, but at higher levels these can also suppress the growth of planktonic cells.

### Effects of Herring Oil, DHA, and EPA on Hemolysis by *S. aureus*

**FIGURE 3:**
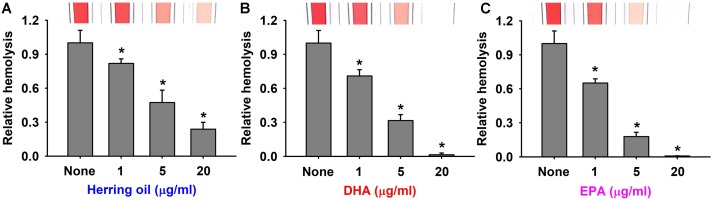
Anti-hemolytic activities of herring oil, DHA, and EPA. Human red blood cell hemolysis by *S. aureus* was investigated in the presence of herring oil **(A)**, DHA **(B)**, or EPA **(C)** after culture for 20 h. The images show hemolysis activities in spectrophotometer cuvettes. ^∗^*p* < 0.05 vs. non-treated controls.

*Staphylococcus aureus* produces α-toxin, which is one of the most investigated *S. aureus* cytotoxins. α-Toxin integrates into the membranes of erythrocytes causing hemolysis (Bhakdi et al., 1984; Song et al., 1996), which contributes to biofilm formation (Caiazza and O’Toole, 2003). Thus, the effects of herring oil, DHA, and EPA on human red blood cell hemolysis by *S. aureus* were investigated. As a control, herring oil, DHA, and EPA without *S. aureus* cells did not show any hemolytic activity (data not shown). Interestingly, all three dose-dependently inhibited the hemolytic activity of *S. aureus* (**Figure 3**), and DHA and EPA at 20 μg/ml completely abolished its hemolytic activity, whereas herring oil at 20 μg/ml reduced its hemolytic activity by >75%. Also, herring oil, DHA, and EPA all significantly reduced the hemolytic activity by *S. aureus* even at 1 μg/ml, which is much lower than the concentrations required for anti-biofilm and antimicrobial activities.

These results support that the inhibitions of *S. aureus* biofilm formation by herring oil, DHA, and EPA are associated with the inhibition of hemolysis, and suggest that the genes related to biofilm formation and hemolysis might be responsible for these events. The present study is the first to report that herring oil, DHA, and EPA suppress the hemolysis of human red blood cells by *S. aureus*, which has important implications regarding the control of *S. aureus* virulence.

### Effects of DHA, EPA, and Herring Oil on *S. aureus* Survival in the Presence of Human Whole Blood

**FIGURE 4:**
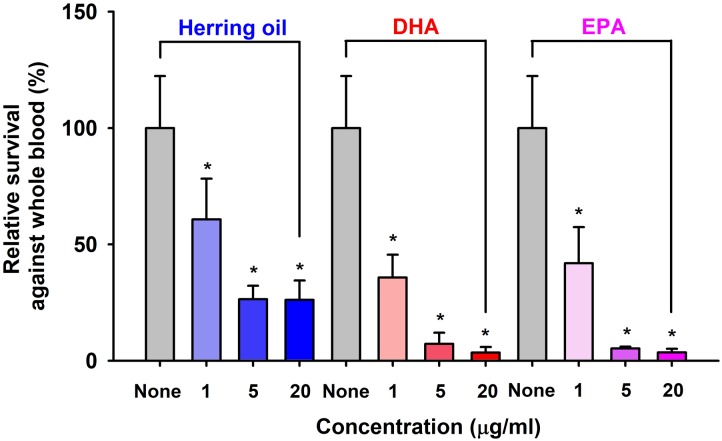
Killing of *S. aureus* by whole blood cells in the presence of herring oil, DHA, or EPA. *S. aureus* cells were cultured in the presence of herring oil, DHA, or EPA at 1, 5, and 20 μg/ml for 20 h, then mixed with freshly drawn human whole blood and cultured for 3 h. Percent survival of *S. aureu*s treated with or without the test compounds is represented. ^∗^*p* < 0.05 vs. non-treated controls.

*Staphylococcus aureus* characteristically exhibits resistance to phagocytes in humans and other animals, and thus, we used a survival test to investigate the effects of herring oil, DHA, and EPA on *S. aureus* survival in the presence of human whole blood. Herring oil, DHA, or EPA at 1, 5, or 20 μg/ml significantly reduced the survival of *S. aureus* exposed to fresh human whole blood (**Figure 4**), indicating that they weaken the ability of *S. aureus* to resist the human innate immune system.

### Effects of DHA, EPA, and Herring Oil on *S. aureus* Virulence in the Nematode Model

**FIGURE 5:**
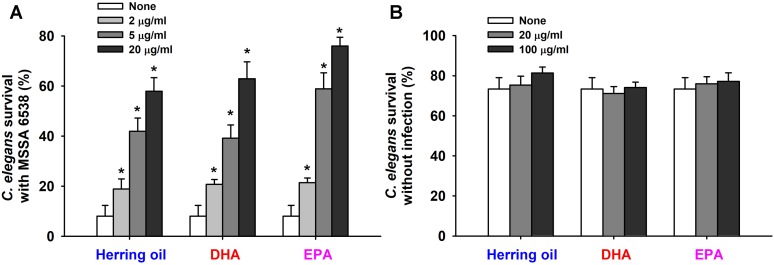
Effects of herring oil, DHA, and EPA on nematode survival. *C. elegans fer-15;fem-1* strain survival after infection with *S. aureus* MSSA 6538 in the presence of DHA, EPA, or herring oil (2, 5, or 20 μg/ml) after exposure for 1 day at 25°C **(A)**. Herring oil, DHA, and EPA toxicities were assessed by determining survival rates versus non-infected nematodes **(B)**. Survival was assessed as dead or alive based on movement. ^∗^*p* < 0.05 versus non-treated controls.

*Staphylococcus aureus* colonizes and replicates in the digestive tract of *C. elegans* and has the ability to kill *C. elegans* by an infection-like process that exhibits remarkable overlap with that observed in mammals (Garsin et al., 2003). Hence, the effects of herring oil, DHA, and EPA on *S. aureus* virulence were investigated by analyzing *C. elegans* survival in the presence of *S. aureus*. Herring oil, DHA, and EPA were found to markedly prolong *C. elegans* survival in the presence of *S. aureus* (**Figure 5A**). For example, nematode survival was only ∼10% in the presence of untreated *S. aureus*, whereas in the presence of herring oil, DHA, or EPA at 20 μg/ml more than 55, 60, and 70%, respectively, of worms survived. In other words, the survival rate of *C. elegans* was increased more than seven and ninefold when worms were exposed to *S. aureus* in the presence of DHA or EPA at 20 μg/ml, respectively (**Figure 5A**). In addition, no toxic effects were observed when non-infected worms were exposed to herring oil, DHA, or EPA at concentrations up to 100 μg/ml (**Figure 5B**).

### Transcriptional Changes Induced by DHA, EPA, and by Herring Oil in *S. aureus*

**FIGURE 6:**
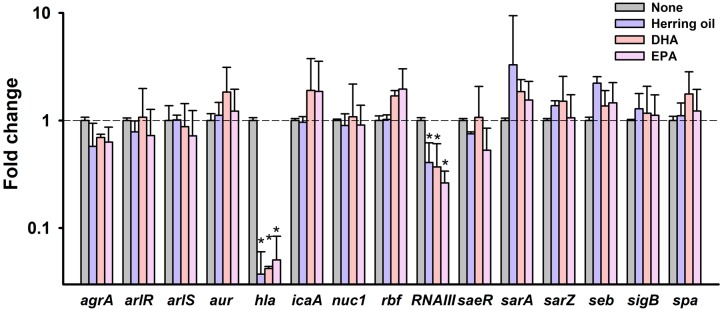
Transcriptional profile changes in *S. aureus* after treatment with herring oil, DHA, or EPA. *S. aureus* MSSA 6538 was incubated in LB broth containing herring oil (100 μg/ml), DHA, or EPA (20 μg/ml) for 5 h with agitation at 250 rpm. Relative transcriptional profiles were determined by qRT-PCR with respect to 16s rRNA expression. qRT-PCR was performed in duplicate and fold-changes were calculated using treated versus untreated *S. aureus*. ^∗^*p* < 0.05 vs. non-treated controls.

To investigate the molecular mechanisms underlying the inhibitory effects of herring oil, DHA, and EPA against *S. aureus*, expressions of selected biofilm- and virulence-related genes, and important regulatory genes were investigated in *S. aureus* cells by qRT-PCR. Herring oil, DHA, and EPA dramatically reduced the expression of α-hemolysin (*hla*) by 27-, 24-, and 20-fold, respectively (**Figure 6**). Also, herring oil, DHA, and EPA downregulated regulatory RNA molecule (*RNAIII*) that is present upstream of the *agr* operon while the expressions of other genes were not affected (**Figure 6**). It has been reported that RNAIII stimulate *hla* translation (Morfeldt et al., 1995). Hence, *hla* repression is partially due to the down-regulation of RNAIII by herring oil, DHA, and EPA.

α-Hemolysin is known to play a critical role in cell-to-cell interactions during biofilm formation (Caiazza and O’Toole, 2003; Anderson et al., 2012; Scherr et al., 2015). This reduction of α-hemolysin expression has been discovered in recently published articles showing that alizarin (Lee et al., 2016), azithromycin (Gui et al., 2014), flavonoids (Cho et al., 2015), norlichexanthone (Baldry et al., 2016), and stilbenoids (Lee et al., 2014) inhibit both biofilm formation and hemolysis by *S. aureus*. Our findings support that α-hemolysin has a positive relationship with biofilm formation in *S. aureus*. In addition, it has been shown *hla* is essential for *S. aureus* pathogenicity in a nematode model (Sifri et al., 2003, 2006). Notably, neither herring oil, DHA, or EPA significantly affected the expressions of the biofilm-related genes namely, *agrA*, *arlR*, *arlS*, *aur*, *icaA*, *nuc1*, *rbf*, *saeR*, *sarA*, *sarZ*, *seb*, *sigB*, or *spa*. Thus, the present study indicates that herring oil, DHA, and EPA attenuate *S. aureus* virulence, as evidenced by reductions in its anti-biofilm (**Figures 1**, **2**) and anti-hemolytic (**Figure 3**) activities, and partially by down-regulating the expression of the *hla* gene (**Figure 6**).

These results indicate that herring oil, DHA, and EPA could be used to treat *S. aureus* infections as lone drug or in combination with traditional antibiotics (**Figure 5**). Furthermore, because all three are considered intrinsically safe in animal, they could be used for medicinal purposes or for example, to surface treat in food processing facilities without undue restriction.

## Conclusion

The DHA and EPA are long chain omega-3 fatty acids, which have major health benefits in man. More specifically, circulating levels of DHA and EPA are essential part of the body’s defense system and play key roles in the inhibitions of inflammation and host immune responses (Calder, 2013). Herring oil was found to contain DHA and EPA at levels of 11.3 and 5.8% by weight, respectively, and inhibit biofilm formation and hemolysis by and the virulence of *S. aureus*. Interestingly, the ability of *S. aureus* to survive exposure to human whole blood was reduced in the presence of DHA or EPA, and exposure to DHA or EPA also increased infected nematode survival. These findings show for the first time that DHA and EPA suppress *S. aureus* biofilm formation and its virulence. Also, herring oil and its constituent omega-3 fatty acids (DHA and EPA) might be useful for the treatment and/or prevention of surface-associated biofilm formation by *S. aureus* (including MRSA strains) and for suppressing its virulence.

## Author Contributions

Y-GK, J-HL, CR, and SO performed the experiments and analyzed the data. Y-GK, J-HL, JP, and JL designed the study and wrote the paper. All authors read and approved the final manuscript.

## Conflict of Interest Statement

The authors declare that the research was conducted in the absence of any commercial or financial relationships that could be construed as a potential conflict of interest.
