# Supplementary material for: Herring Oil and Omega Fatty Acids Inhibit Staphylococcus aureus Biofilm Formation and Virulence
Source: Front Microbiol. 2018 Jun 15;9:1241. doi: 10.3389/fmicb.2018.01241 (PMC6014104; doi:10.3389/fmicb.2018.01241)
Supplement: Supplementary file 1 [file Table_1.docx]

**Supplementary material**

**Supplementary Table 1.** Sequences of the primers used for quantitative RT-PCR.

| Gene | Name | Primer |
| --- | --- | --- |
| *16S*  *rRNA* | A component of ribosomes | Forward 5'-TGT TTG ACG ATG TTT GAG CA-3' |
|  |  | Reverse 5'-CCT TCC TCC AGT TCA GAT GC -3' |
| *agrA* | Quorum-sensing regulator A | Forward 5'-TGA TAA TCC TTA TGA GGT GCT T-3' |
|  |  | Reverse 5'-CAC TGT GAC TCG TAA CGA AAA-3' |
| *arlR* | Response regulator | Forward 5'-TTA CGG TGC AGG CGA TTA TAT AG-3' |
|  |  | Reverse 5'-TAC CGT TGA CAT CGA TAA TAT CC-3' |
| *arlS* | Histidine-protein kinase | Forward 5'-TGG AAT ACC AAT TCC ATG ATC T-3' |
|  |  | Reverse 5'-TGC AAT CAA ATA TGA TGT GAA GAA-3' |
| *aur* | Zinc metalloproteinase aureolysin | Forward 5'-ACC GTG TGT TAA TTC GTG TGC TA-3' |
|  |  | Reverse 5'-ATG GTC GCA CAT TCA CAA GTT T-3' |
| *hla* | α-Hemolysin | Forward 5'-CGG CAC ATT TGC ACC AAT AAG GC-3' |
|  |  | Reverse 5'-GGT TTA GCC TGG CCT TCA GC-3' |
| *icaA* | Intercellular adhesion A | Forward 5’-TGA ACC GCT TGC CAT GTG-3’ |
|  |  | Reverse 5’-CAC GCG TTG CTT CCA AAG A-3’ |
| *nuc1* | Nuclease | Forward 5'-CAC CTG AAA CAA AGC ATC CTA A-3' |
|  |  | Reverse 5'-TAT ACG CTA AGC CAC GTC CAT-3' |
| *rbf* | Regulator of biofilm formation | Forward 5'-TTA GAA GGA ATC TTT AAA ACC TTA TTG AAT AA-3' |
|  |  | Reverse 5'-TTG TGA ATT TTT CTT CTT CGG ACA-3' |
| *RNAⅢ* | Transcriptional regulator | Forward 5'-ATC GAC ACA GTG AAC AAA TTC AC-3' |
|  |  | Reverse 5'-CTC TAC TAG CAA ATG TTA CTC AC-3' |
| *saeR* | Response regulator | Forward 5'-GCC TTA ACT TTA GGT GCA GAT GAC TAT GTC-3' |
|  |  | Reverse 5'-CGA CAG TTG TTC AAC TGG TTG ATG ATG G-3' |
| *sarA* | Transcriptional regulator | Forward 5'-GAG TTG TTA TCA ATG GTC-3' |
|  |  | Reverse 5'-GTT TGC TTC AGT GAT TCG-3' |
| *sarZ* | HTH-type transcriptional regulator | Forward 5'-CCT ATA CTG GTT ACA TTG TTT TAA TGG-3' |
|  |  | Reverse 5'-TGG TGT CAG TGT TCC AGA ATC-3' |
| *seb* | Enterotoxin B | Forward 5'-TGT TCG GGT ATT TGA AGA TGG -3' |
|  |  | Reverse 5'-CGT TTC ATA AGG CGA GTT GTT-3' |
| *sigB* | RNA Polymerase sigma factor | Forward 5'-AAG TGA TTC GTA AGG ACG TCT-3' |
|  |  | Reverse 5'-TCG ATA ACT ATA ACC AAA GCC T-3' |
| *spa* | Protein A | Forward 5'-ACC AGA AAC TGG TGA AGA AAA TCC-3' |
|  |  | Reverse 5'-TAA CGC TGC ACC TAA GGC TAA TG-3' |
